# Hepatitis B virus downregulates vitamin D receptor levels in hepatoma cell lines, thereby preventing vitamin D-dependent inhibition of viral transcription and production

**DOI:** 10.1186/s10020-018-0055-0

**Published:** 2018-10-16

**Authors:** Neta Gotlieb, Irena Tachlytski, Yelena Lapidot, Maya Sultan, Michal Safran, Ziv Ben-Ari

**Affiliations:** 1 Liver Reaserch Laboratory, Sheba Medical Center, Tel Hashomer, 52620 Ramat Gan, Israel; 2 Liver Disease Center, Sheba Medical Center, Tel Hashomer, 52620 Ramat Gan, Israel; 3 The Sackler School of Medicine, Tel Aviv University, Tel Aviv, Israel

**Keywords:** Vitamin D, Hepatitis B virus (HBV), Vitamin D receptor (VDR), Immune system, Downregulation

## Abstract

**Background:**

Vitamin D is a key immune-modulator that plays a role in the innate and adaptive immune systems. Certain pathogens impair the immune defense by downregulating the vitamin D receptor (*VDR*) pathway. Low serum levels of vitamin D are associated with increased hepatitis B virus (HBV) replication. Our study aimed to assess the in-vitro relationship between HBV production and Vitamin D signaling pathway and to explore the associated mechanism(s).

**Methods:**

HBV transcription and replication was evaluated by qRT-PCR of the HBV-RNA and covalently closed circular DNA (cccDNA). Furthermore, we have transfected the 1.3 X HBV-Luc plasmid to the cells and measured the Luciferase activity using Luminometer. Vitamin D signaling pathway activation was evaluated by measuring the expression levels of *VDR*, *CYP24A1*, Tumor necrosis factor α (TNF*α*) and cathelicidin (*CAMP*) by qRT-PCR. All assays were performed on HepG2.2.15, HepG2, and HepAD38 cells treated with or without Vitamin D active metabolite: calcitriol.

**Results:**

Calcitriol did not suppress HBV transcription, cccDNA expression or HBV RNA levels in HepG2.2.15 cells. However, *VDR* transcript levels in HepG2.215 cells were significantly lower compared to HepG2 cells. Similar results were obtained in HepAD38 cell where *VDR* expression was down-regulated when HBV transcript level was up-regulated. In addition, calcitriol induced *VDR*-associated signaling, resulting in upregulation of *CYP24A1*, *TNFα* and *CAMP* expression level in HepG2 cells but not in the HepG2.2.15 cells.

**Conclusions:**

These findings indicate that *VDR* expression is downregulated in HBV-transfected cells, thereby preventing vitamin D from inhibiting transcription and translation of HBV in vitro. HBV might use this mechanism to avoid the immunological defense system by affecting both *TNFα* and *CAMP* signaling pathways.

**Electronic supplementary material:**

The online version of this article (10.1186/s10020-018-0055-0) contains supplementary material, which is available to authorized users.

## Background

Hepatitis B virus (HBV) infection is a global public health problem, estimated to affect approximately 2 billion people, of whom, 240 million are chronic carriers. About 20–30% of HBV carriers will progress to liver failure, hepatocellular carcinoma (HCC) and eventually, liver transplantation (Seeger & Mason, 2000). Current antiviral HBV therapy regimens dramatically decrease the viral load and thereby inhibit disease progression and complications. However, they do not bring to complete viral clearance in the infected hepatocytes, probably due to the synthesis of nuclear covalently closed circular DNA (cccDNA) and its integration into the hepatocyte genome (Ahmed et al., 2015). Thus, contemporary antiviral treatment (Marcellin et al., 2013) do not fully eliminate the likelihood of progression to cirrhosis and HCC.

Vitamin D exhibits extra-skeletal functions such as immune response, insulin secretion and cellular division (Deluca & Cantorna, 2001; Vanherwegen et al., 2017). Furthermore, Vitamin D deficiency is associated with an increased risk for various autoimmune disorders (Barbalho et al., 2017; Hassanalilou et al., 2017; Knutsen et al., 2017; Sandhya et al., 2017), cancer (Heidari et al., 2017; Hu et al., 2017; Hohaus et al., 2017), metabolic disorders (Wojcik et al., 2017; Schmitt et al., 2018; Lim et al., 2017; Chen et al., 2017)as well infections caused by influenza, rhinovirus, respiratory syncytial virus (RSV) and Human Immunodeficiency Virus (HIV) with high mortality rate from these pathogens (Borella et al., 2014; Pletz et al., 2014; Orkin et al., 2014; Watkins et al., 2015). Calcitriol, the active metabolite of Vitamin D, binds the nuclear Vitamin D receptor (*VDR*), which is responsible for the biological activity of vitamin D in the cell. *VDR* is found in a diverse range of tissues, including the liver and immune cells, such as T cells, monocytes and macrophages. After binding its ligand, *VDR* forms a heterodimer with the X receptor, which binds to vitamin D response elements present on target genes. The complex elicits an extensive biological response via regulation of gene transcription and stimulation of intra-cellular signaling pathways. Evidence of a crucial role played by Vitamin D in defending the body from microbe invasion has recently emerged. It was shown that Vitamin D can induce the expression of antimicrobial peptides (also known as host defense peptides), such as cathelicidin (*CAMP*), which have been demonstrated to disrupt the integrity of the microbe membrane, resulting in its death (Gombart, 2009). Furthermore, Vitamin D has also been shown to stimulate the expression of several cytokines, such as Tumor Necrosis Factor α (TNFα) (Golovko et al., 2005), that regulate both the recruitment of inflammatory cells to the area of infection and the activation of macrophage and T cell functions. Certain pathogens such as Mycobacterium Tuberculosis and HIV-1, can impair the innate immune defenses by downregulating the VDR pathway (Haug et al., 1994; Haug et al., 1998; Huang et al., 2015). Additionally, a recent meta-analysis showed that VDR polymorphism increases the risk for HBV infection (He et al., 2017).

Studies have shown a high incidence (50–90%) of vitamin D deficiency in patients with chronic liver disease, mainly, nonalcoholic fatty liver disease, cirrhosis and chronic hepatitis C infection. In vitro, vitamin D (3) showed remarkable antiviral activity by inhibiting hepatitis C virus (HCV) production in Huh7.5 hepatoma cells, suggested to be mediated by its active metabolite, calcitriol. Supplementing antiviral treatment in HCV patients with vitamin D significantly increased the odds for cure (sustained virologic response SVR) in patients with HCV genotypes 1, 2 and 3 and in post-transplantation patients (Gutierrez et al., 2011; Villar et al., 2013; Abu-Mouch et al., 2011; Nimer & Mouch, 2012; Bitetto et al., 2011; Kim et al., 2017).

In sharp contrast to HCV, the relationship between vitamin D metabolism and HBV infection is largely elusive. Chan et al. noted a high prevalence of abnormally low vitamin D levels among untreated, active chronic hepatitis B (CHB) patients (Chan et al., 2015). Similarly, in their prospective cohort study, Wong et al. also concluded that vitamin D deficiency is common among patients with CHB and is associated with adverse clinical outcomes, including HCC and increased rated of liver-related deaths (Wong et al., 2015). Farnik et al. (Farnik et al., 2013) demonstrated a correlation between low serum vitamin D levels in chronic HBV patients and high viral replication. Additionally, chronic HBV increased the risk of vitamin D deficiency. However, the researchers failed to detect serum HBsAg, which have been shown to reflect active intrahepatic cccDNA (Martinot-Peignoux & Marcellin, 2016). A recent clinical study found that following long-term treatment with nucleoside/nucleotides analogues the mean level of 25(OH)D3 increased significantly in patients with undetected levels of HBV-DNA (Chen et al., 2015). The current study aimed to determine the relationship between the vitamin D pathway and HBV transcription and replication in vitro.

## Methods

### Reagents

Calcitriol was purchased from Sigma (St. Louis, MO, USA).

### Cell culture and treatment

HepG2 (hepatoma) cell line and HepG.2.215 (HBV-infected hepatoma cells) were generous gift from the lab of Prof. Shaul, Weizmann Institute of Science in Rehovot, Israel. These cells were maintained in Dulbecco’s modified Eagle’s minimal essential medium (Biological Industries, Israel), as previously described (Rechtman et al., 2010). Cells were grown to reach near confluence 24 h prior to transfection, which was carried out using the Lipofectamin 2000 reagent (Invitrogen Carlsbad, California USA), according to the manufacture’s instructions.

HepAD38 cells were generous gift from the lab of Prof. Seeger, Fox Chase Cancer Center, PA USA, and David Durantel Cancer Research Center of Lyon, France. These cells were cultured in a Dulbecco’s modified Eagle’s minimal essential medium with 10% FCS with or without 0.3 μg/mL tetracycline (Sigma St. Louis, MO, USA) for 7 days before analyzing the cells.

All cell lines were treated with increasing concentrations of Calcitriol (0–100 nM) (Sigma St. Louis, MO, USA) for 24 h.

### Plasmids

The previously described 1.3 X HBV-Luc plasmid (Rechtman et al., 2010), was a generous gift from the lab of Prof. Shaul, Weizmann Institute of Science in Rehovot, Israel.

### Luciferase genetic reporter assays

Luciferase assay was performed using the Dual Luciferase Assay System (Promega Madison, WI, USA), according to the manufacturer’s instructions. The luminescence levels were determined using a Berthold Technologies luminometer (Titertek-Berthold, Pforzheim,Germany).

### RNA purification and quantitative real-time PCR analysis

Total RNA was extracted using TRI-reagent (Sigma St. Louis, MO, USA), followed by treatment with 1 U RNase-free DNase (Roche). Reverse transcription was performed on 2 μg total RNA, using the High Capacity cDNA Reverse Transcription Kit (Applied Biosystems Carlsbad, CA USA), according to the manufacturer’s instructions. qRT-PCR was performed on 50 ng cDNA samples, using the SYBR Green Real-Time PCR Kit (Applied Biosystems Carlsbad, CA USA) according to manufacturer’s specifications, with gene-specific primers and HPRT as the reference endogenous control (Additional file 1: Table S1).

All reactions were performed in triplicates and relative gene expression was determined using the 2.δδCt method with ABI Prism 7000 SDS (Applied Biosystems Carlsbad, CA USA).

### DNA purification and measurement of cccDNA levels

DNA was extracted using the Q/Amp DNA Mini-kit according to the manufacturer’s instructions (QIAGEN Valencia, CA USA). cccDNA expression levels were measured by qRT-PCR analysis (Additional file 1: Table S1).

### Western blot analysis

Proteins were extracted using RIPA extraction buffer (Sigma, St. Louis, MI, USA) containing complete, mini-protease inhibitor cocktail tablets (Roche Basel, Switzerland) and phosphatase inhibitors (coktail2&3)(Sigma St. Louis, MO, USA). Protein levels were quantified using a commercial BCA kit (Pierce Appleton, WI USA). Liver protein extracts (40 μg protein/ lane) were separated under reducing conditions on polyacrylamide gels by SDS-PAGE, and then transferred to nitrocellulose membranes. Membranes were soaked for 1 h in a blocking solution, comprised of phosphate buffer saline (PBS), 5% non-fat milk and 0.01% (*v*/v) Tween-20 (Sigma), and then incubated with anti-VDR antibodies (1:300) (Santa Cruz Dallas, TX USA.) for 1–2 h, at RT. After incubation, the membrane was washed three times with PBST, and then exposed to goat anti-mouse horse radish peroxidase-conjugated antibodies (1:5000 in PBST) (Jackson West Grove, PA, USA) for an additional 1 h. Antibody-antigen complexes were visualized by ECL on an X-ray film.

### Statistics

All experiments were done at least three times. Error bars in the graphs present the calculations of standard deviation. Differences between two groups were calculated using 2 tales ttest. Significant result (p) is calculated as < 0.05. Differences between more than two groups were calculated using ANOVA. F represents the statistical result of the ANOVA test. P represent a statistical difference if < 0.05.

## Results

### HBV transcription, expression and cccDNA levels are not affected by calcitriol treatment

**Fig. 1:**
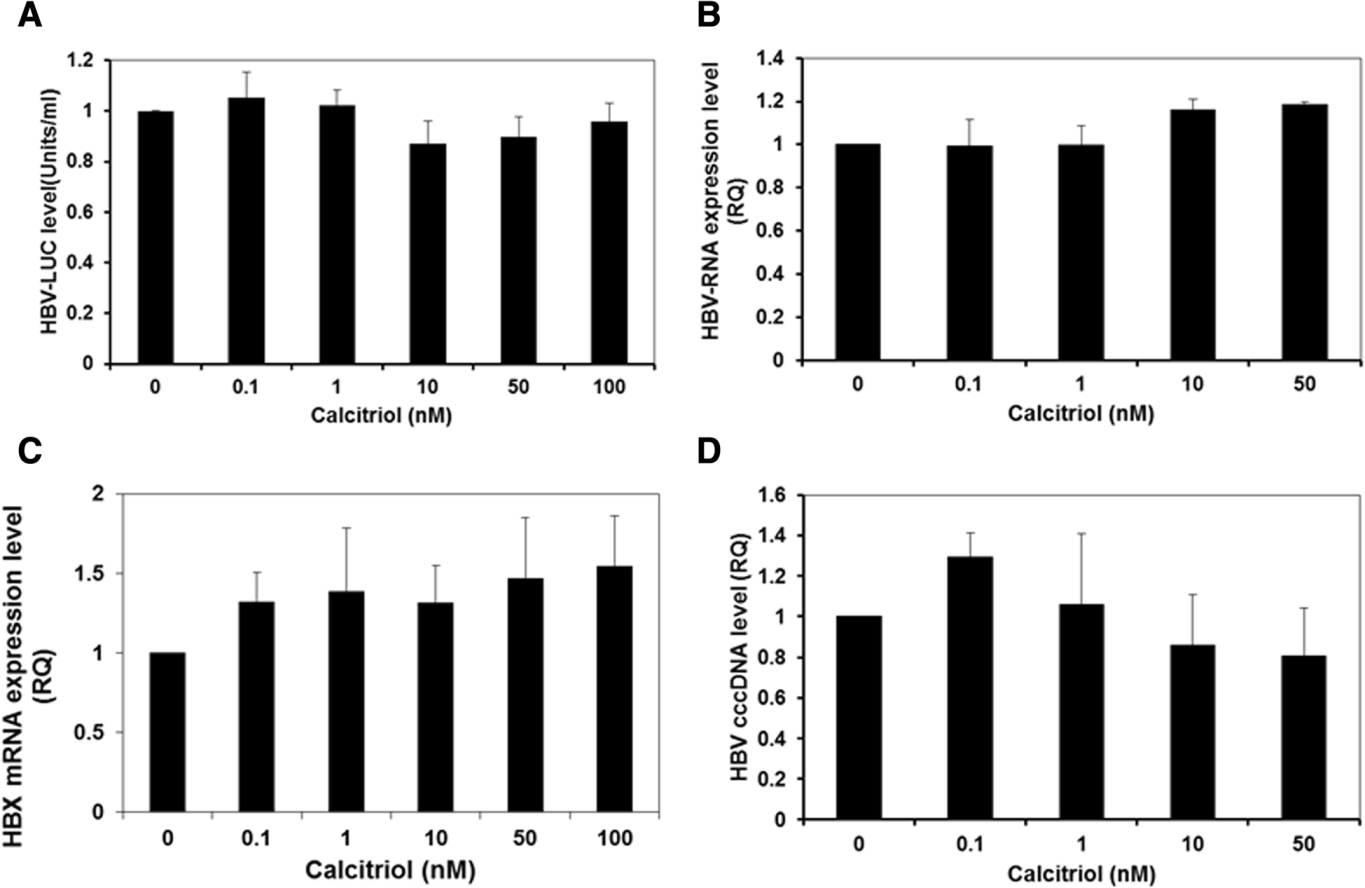
Treatment with increasing concentrations of Calcitriol does not affect the transcription and expression levels of HBV genes and cccDNA. HepG2 cells were transfected with the 1.3 X HBV-Luc plasmid. 24 h later, the cells were treated with increasing concentrations of Calcitriol or EtOH, as a control. After an additional 24 h, the cells were harvested and analyzed for Luciferase activity (**a**). HBV-RNA (**b**), HBX (**c**) and cccDNA (**d**) were measured using RT-PCR. Results are expressed as mean ± standard deviation

In order to assess the effect of Calcitrol on the expression levels of HBV, HepG2 cells were transfected with the 1.3 X HBV-Luc plasmid HBV construct containing a luciferase ORF under the HBV core promoter. 24 h after the transfection cells were treated with increasing concentrations of Calcitriol for additional 24 h and the levels of Luciferase activity were measured. Treatment with increasing concentrations of Calcitriol did not alter the expression of HBV as demonstrated by the levels of luciferase activity (Fig. 1a (f = 0.937 *p* = 0 .491). Furthermore, when we have measured the HBV-RNA (B) levels and HBX (C) levels after 24 h of treatment with increasing concentrations of Calcitriol, we found no differences in the levels of these molecules (HBV-RNA,f = 2.364 *p* = 0.103 HBX f = 0.440 *p* = 0.815).

In order to further test the functionality of the ongoing viral replication, we then measured the expression level of cccDNA in these HBV-expressing cells. Treatment with increasing concentrations of Calcitriol did not significantly affect cccDNA, expression levels (Fig. 1d) (P=NS).

### VDR transcripts and protein expression levels are repressed by HBV

**Fig. 2:**
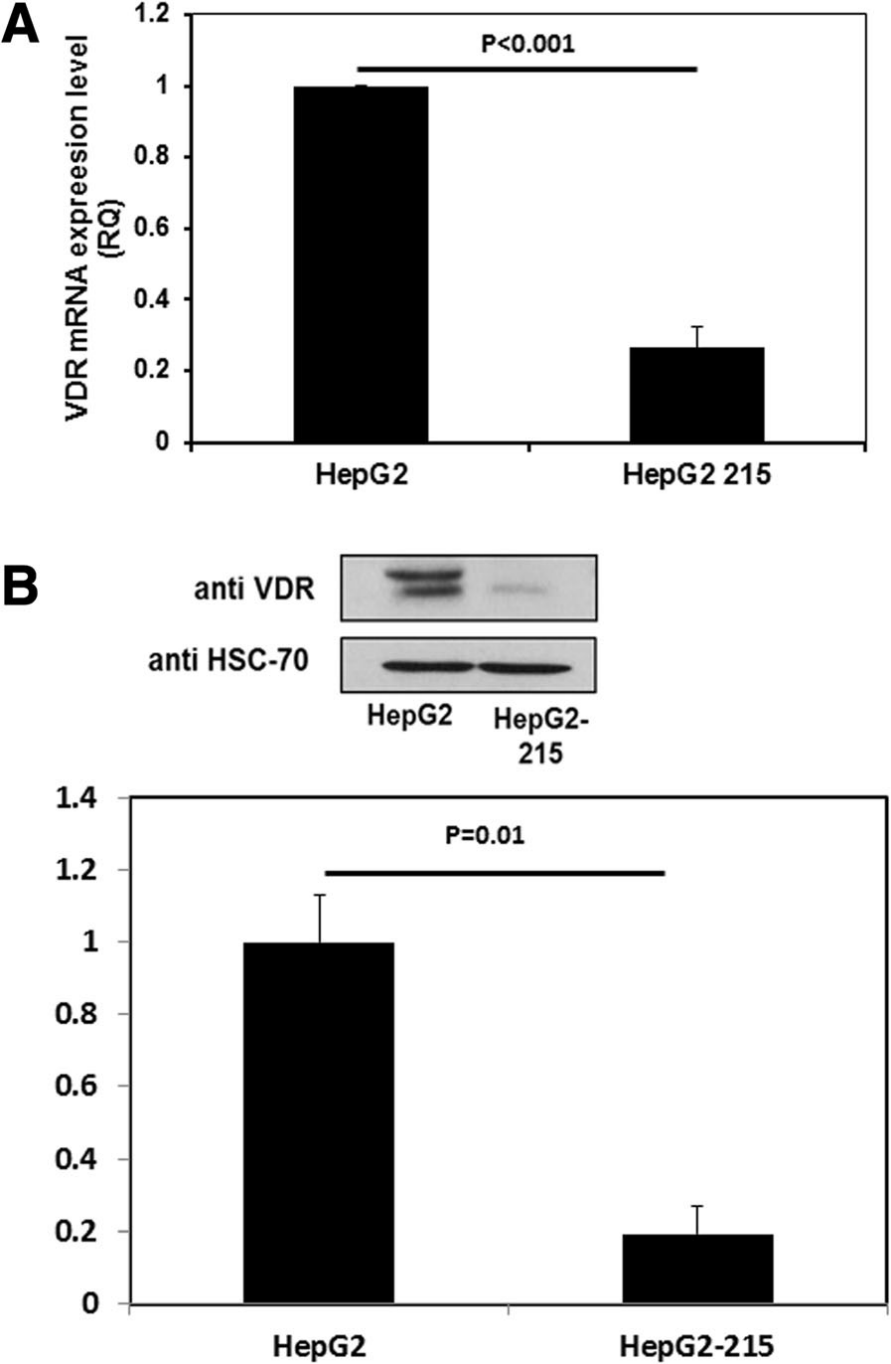
VDR transcript and protein levels were lower in HepG2–2.15 compared to HepG2 cells. HepG2.2.15 and HepG2 cells were harvested and the levels of VDR RNA (**a**) and protein (**b**) were measured using RT-PCR and western blot analysis, respectively (*p* < 0.001). The western blot presented in the figure is a representative of three different experiments; all of these experiments were calculated in the quantification. Results are expressed as mean ± standard deviation

Since our results demonstrated that vitamin D does not affect HBV levels, we than set to investigate the influence of HBV on the vitamin D pathway. Therefore, we set to compare the vitamin D receptor levels in HepG2.2.15 (HBV-expressing cells) versus HepG2 (non-transfected) cells. VDR transcript and protein levels were significantly lower (*p* < 0.001) in the HBV-infected hepatocytes compared with HepG2 cells (Fig. 2a and b).

### HBV represses the expression of VDR in infected cells

**Fig. 3:**
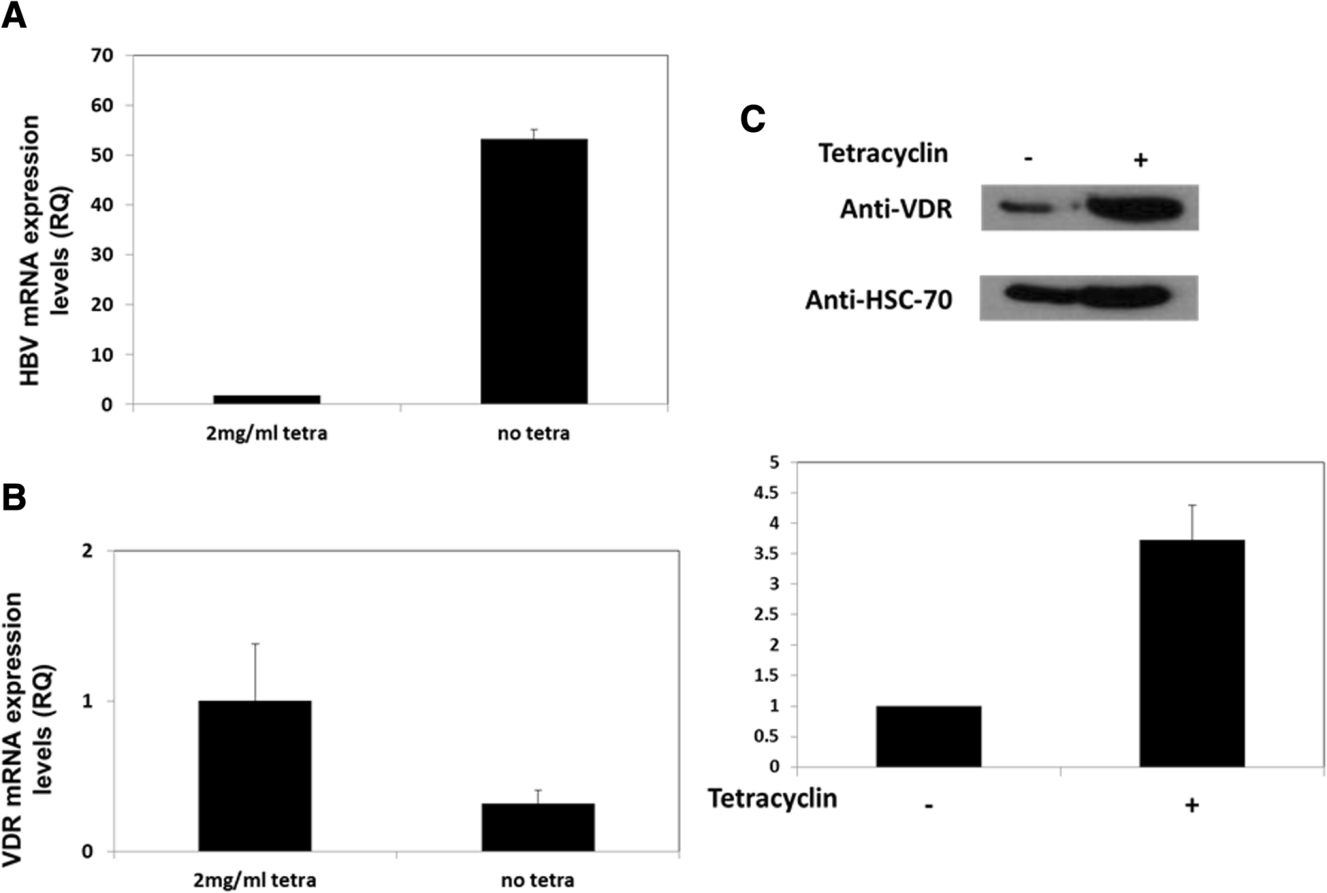
VDR transcript levels after the induction of HBV expression in HepAD38 cells. HepAD38 cell were cultured in the presence of 0.3 μg/ml tretracyline. In the next stage, HepAD38cells were washed and the medium was replaced by tetracycline free medium. HBV (**a**) and VDR (**b**) transcripts levels were measured using RT-PCR. VDR protein levels (**c**) were measured using western blot analysis. The western blot presented in the figure is a representative of three different experiments; all of these experiments were calculated in the quantification. Results are expressed as mean ± standard deviation

Since HepG2 and HepG2.2.15 are two distinct cell lines although they have the same origin, some of the alterations in their transcriptome might not be due to the HBV expression. Hence, we decided to validate these results in another cellular model; HepAD38 cell line. HepAD38 cells, a variant of HepG2 cells, express the HBV genome under the control of a tetracycline. In the presence of the antibiotic, HepAD38 is free of virus due to the repression of pregenomic (pg) RNA synthesis. Upon removal of tetracycline from the culture medium, HepAD38 express viral pg RNA (Ladner et al., 1997). Therefore, HepAD38 cells were cultured in a medium free of tetracyclin for 7 days and levels of HBV and VDR transcripts were measured. As expected HBV transcript level were up-regulated after tetracycline withdrawal (Fig. 3a). However a significant decrease in the levels of VDR was noted (Fig. 3b) (*p* < 0.05). Measuring the levels of the VDR proteins in those cells with or without tetracycline showed us again lower levels of VDR protein in the cells that express the HBV virus (Fig. 3c).

### VDR-induced CYP24A1 expression in calcitriol-treated HepG2 and HepG2.2.15 cells

**Fig. 4:**
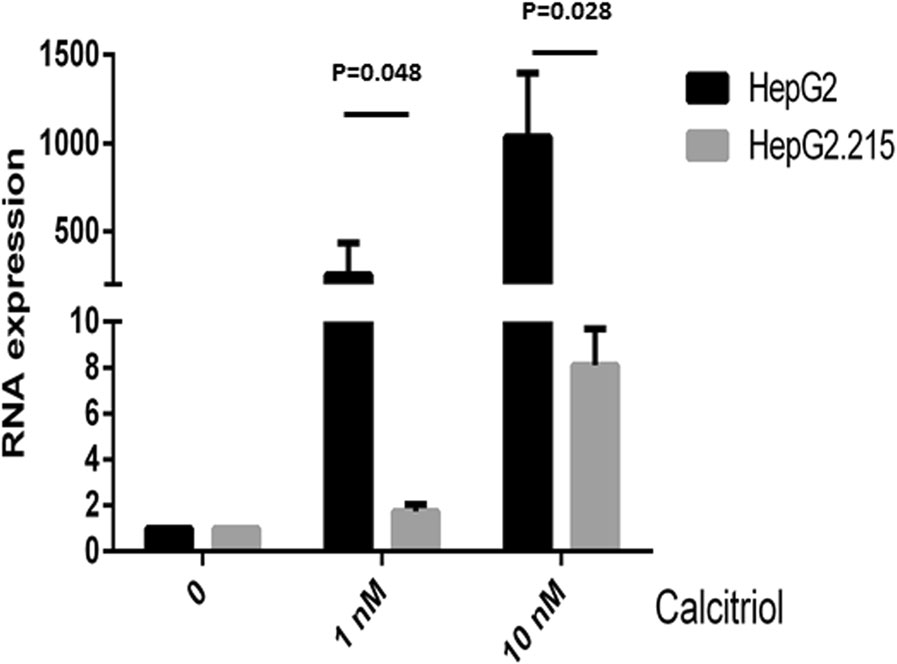
Up-regulation in CYP24A1 expression level as a result of calcitriol treatment is attenuated in HBV-transfected cells. HepG2.2.15 and HepG2 cells were treated with either 1 nM or 10 nM of Calcitriol or with EtOH, as a control. 24 h later, the cells were harvested and CYP24A1 transcript levels were measured using RT-PCR. (HepG2 vs HepG2 HepG2.2.15 (1 nM) *p* = 0.048, HepG2 vs HepG2 HepG2.2.15 10 nM *p* = 0.028). Results are expressed as mean ± standard deviation

CYP24A1 is a known regulator of Vitamin D activity. However, CYP24A1 expression is induced by 25(OH)D3 through activation of the VDR. Since HBV-transfected cells do express lower levels of VDR, we have expected that these cells are unable to activate the Vitamin D signal transduction pathway. Therefore, we have measured the expression level of CYP24A1, following 24 h of treatment with increasing concentrations of Calcitriol. In this experiment we have found, as expected, that the expression levels of CYP24A1were significantly higher in HepG2 compared to HepG2.2.15 after treatment with calcitriol (Fig. 4). CYP24A1 transcript levels increased 1036 fold in calcitriol (10 nM)-treated HepG2 cells, compared to only 8-fold in the calcitriol-treated HepG2.2.15 cells.

### TNFα expression in HepG2.2.15 and HepG2 cells following treatment with calcitriol

**Fig. 5:**
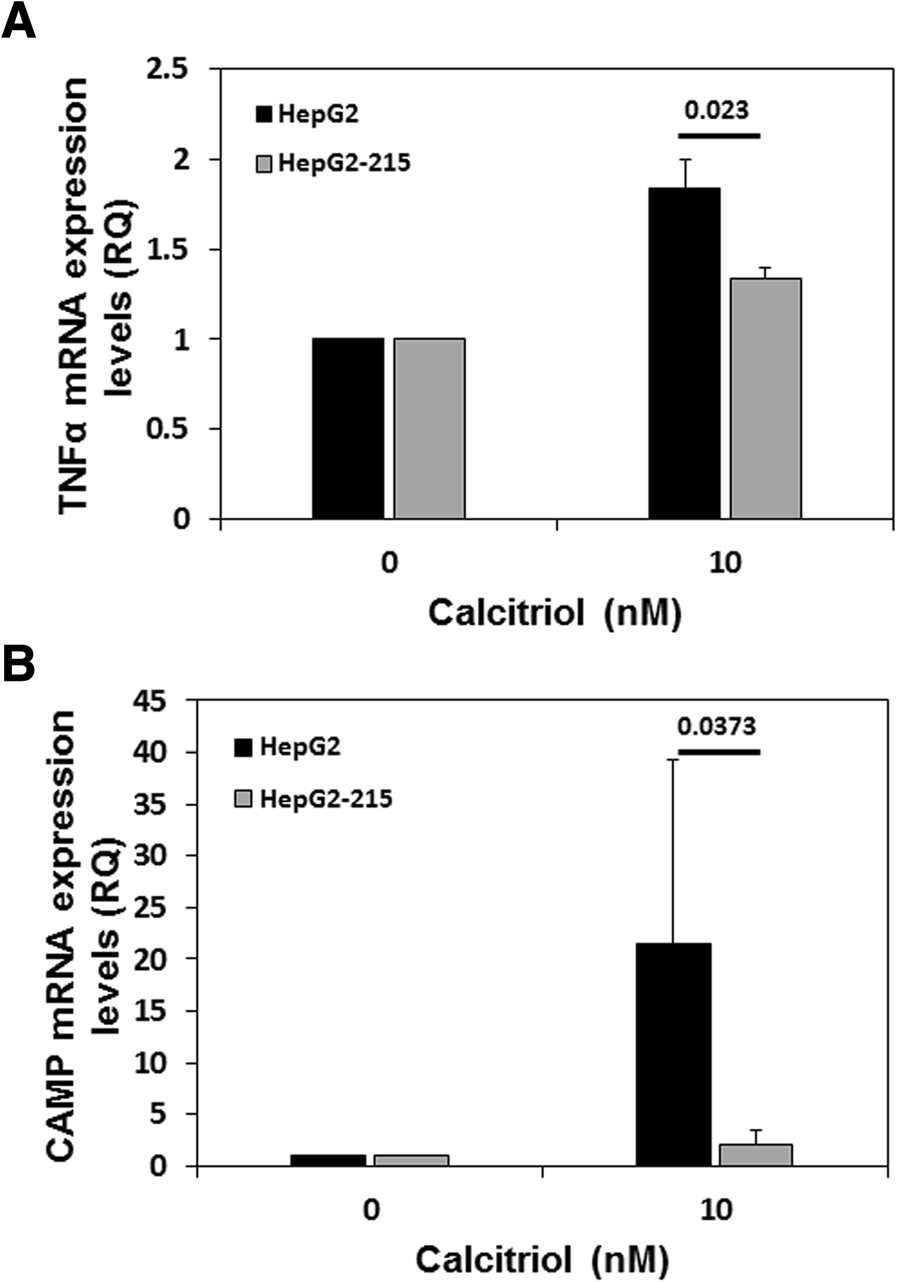
TNFα and CAMP expression levels were significantly lower in Calcitriol-treated HepG2 HepG2.2.15 as compared with HepG2 cells. HepG2.2.15 and HepG2 cells were treated with 10 nM Calcitriol or EtOH, as a control. 24 h later, the cells were harvested and TNFα (**a**) and CAMP (**b**) levels were measured by RT-PCR. (*p* = 0.023 and *p* = 0.0373, respectively). Results are expressed as mean ± standard deviation

As an immune-modulator, Vitamin D can upregulate the expression of several proteins that play a role in the body’s response to microbe invasion, including CAMP, an antimicrobial peptide, and the cytokine TNFα (Gombart, 2009; Golovko et al., 2005). To this end, we analyzed the relationship between the downregulation of VDR in Calcitriol-treated HBV-expressing cells and the activation of CAMP and TNFα. Levels of both TNFα and CAMP transcripts were significantly lower in the HepG2.2.15 compared to HepG2 cells (Fig. 5, *p* = 0.023 and *p* = 0.0373, respectively) following 24 h of treatment with calcitriol.

## Discussion

This study established the relationship between the vitamin D molecular pathway and HBV transcription and replication in vitro. While Calcitriol treatment did not suppress HBV transcription, cccDNA expression and HBV RNA levels in HepG2.2.15 cells, the levels of VDR transcripts in HepG2.2.15 cells was significantly lower compared with non-transfected HepG2 cells. A similar effect was further established using HepAD38 cells where high levels of HBV expression was associated with a decrease in the levels of VDR transcripts and vise versa. Moreover, following the administration of Calcitriol, the expression levels of CYP24A1, an VDR-regulated gene, was significantly lower HepG2.2.15 as compared to HepG2 cells. Finally, the calcitriol-induced VDR signaling pathway, as determined by TNFα and CAMP transcripts levels, was not observed in HepG2.2.15 cells, while significant upregulation was noted in HepG2 cells.

Recently studies (Chan et al., 2015; Wong et al., 2015; Chen et al., 2015) demonstrated a correlation between low serum vitamin D levels in chronic HBV patients and high levels of viral replication. These studies raised the possibility that vitamin D levels inhibit HBV replication. In contrast, our in-vitro study showed that Vitamin D does not affect the rate of HBV replication, and downregulates VDR levels in the presence of the virus, thereby attenuating vitamin D signal transduction.

Vitamin D plays a crucial role in the regulation of genes central to protection against microbe invasion, such as the induction of the expression of antimicrobial peptides (also known as host defense peptides) such as CAMP and defensin. These peptides were demonstrated to disrupt the integrity of the microbe membrane, resulting in its death (Gombart, 2009). In addition, Vitamin D regulates the immune system by managing the expression of TNFα (Golovko et al., 2005), one of the most important pro-inflammatory and pro-immune cytokines. Therefore, downregulation of the vitamin D signaling pathway by viruses, can result in decreased production of antimicrobial peptides and cytokines and as a result, to attenuation of the immune response. Several studies have previously indicated that certain viruses can inhibit the Vitamin D signal transduction. In 2009, Yenamandra et al. demonstrated that VDR mRNA and protein levels were lower in EBV-transformed cells compared with primary B cells (Yenamandra et al., 2009). A few years earlier, Haug et al. reported a marked decrease in serum Calcitriol levels in human immunodeficiency virus (HIV)-infected patients, that correlated with the degree of immunodeficiency and patient survival (Haug et al., 1994). Therefore, in this study, we compared the activation of both TNFa and CAMP in HepG2 cell versus HepG2.2.15 cells following Cacitriol stimulation. Indeed, while the addition of Calcitriol upregulates both CAMP and TNFα expression in HepG2 cells, significantly less transcription of these genes was observed in HepG2.2.15 cells. These findings suggest that HBV can repress the activation of the immune system by downregulating the vitamin D signaling pathway.

Several signaling pathways may be involved in the inhibition of VDR expression following HBV infection. HBx is a 17-kD protein encoded by the X open reading frame of HBV, that complexes with cellular proteins and transactivates virus gene expression and replication (Keasler et al., 2007). Furthermore, HBx protects virus-infected cells from immune-mediated destruction (Arzumanyan et al., 2013). However, we did not detect a correlation between HBx expression level and the administration of calcitriol. Moreover, a relationship between HBx and Vitamin D is yet to be established. Alternatively, HBV polymerase may be involved in the inhibition of VDR expression (Wu et al., 2007). Further studies will be necessary to identify the factors inhibiting VDR expression following viral infection.

## Conclusions

In this study, we have shown that HBV downregulates the expression levels of Vitamin D receptor in the HBV-infected HepG2 cell line, thereby preventing the effect of Vitamin D on viral transcription and production. Furthermore, these findings suggest that HBV might use this mechanism to avoid the immunological defense system, by affecting the expression of immune-modulators such as CAMP and TNFα. The precise mechanisms regulating the innate and adaptive immune response in these cells remain to be further investigated.

## Additional file

Additional file 1: **Table S1.** Primers for RT-PCR. (DOCX 14 kb)

## Abbreviations

cccDNA: Closed circular DNA
HBV: Hepatitis B virus
HCC: Hepatocellular carcinoma
HCV: Hepatitis C virus
HIV: Human immunodeficiency virus
RSV: Respiratory syncytial virus
TNFα: Tumor necrosis factor α
VDR: Vitamin D receptor
