# Supplementary material for: Hepatitis B virus downregulates vitamin D receptor levels in hepatoma cell lines, thereby preventing vitamin D-dependent inhibition of viral transcription and production
Source: Mol Med. 2018 Oct 16;24:53. doi: 10.1186/s10020-018-0055-0 (PMC6192355; doi:10.1186/s10020-018-0055-0)
Supplement: Supplementary file 1 — Table S1. Primers for RT-PCR. (DOCX 14 kb) [file 10020_2018_55_MOESM1_ESM.docx]

Additional file 1: Table S1. Primers for RT-PCR

|  | Forward | Reverse |
| --- | --- | --- |
| HBV | TGTGGATTCGCACTCCTCCAGC | TGCGAGGCGAGGGAGTTCTT |
| HBX | GGACTCTCAGCAATGTCAACGA | AACCTAATCTCCTCCCCCAACT |
| CYP24A1 | GCTGCACAAGAGCCTCAACA | CTGCACTAGGCTGCTGAGAATACT |
| VDR | CCTTCAGGGATGGAGGCAAT | GCAGCCTTCACAGGTCATAGC |
| TNFα | CCCAGGGACCTCTCTCTAATCA | GGCCCGGCGGTTCAG |
| CAMP | TCACCAGAGGATTGTGACTTCAA | TGAGGGTCACTGTCCCCATAC |
| HPRT1 | TGACACTGGCAAAACAATGCA | GGTCCTTTTCACCAGCAAGCT |
| cccDNA | TCCCCGTCTGTGCCTTC | CCCCAAAGCCACCCAA |
| HPRT DNA | CATAGTCTTTCCTTGGGTGTGTTAAA | CCTTTTCACCAGCAAGCTGTTA |
